# The community uptake of an online CRC risk assessment and its utility to assess for a potential hereditary colon cancer syndrome

**DOI:** 10.1186/1897-4287-9-S1-P4

**Published:** 2011-03-10

**Authors:** Carol A Burke, Brandie Leach, Jie Dai, Nandan Patibandla, Elena Manilich, Margaret O'Malley, Lisa LaGuardia, Rocio Lopez, James Church

**Affiliations:** 1 The Sanford R. Weiss, M.D. Center for Hereditary Colorectal Neoplasia, Digestive Disease Institute, 9500 Euclid Ave., Cleveland Clinic, Cleveland, Ohio, 44195, USA

## Purpose

The identification of individuals with Hereditary Non Polyposis Colorectal Cancer (HNPCC) in the population is suboptimal. Causes include lack of physician recognition or failure to take an accurate family history. While colorectal cancer (CRC) in HNPCC is preventable by annual colonoscopy it is underutilized in part by lack of physician recommendation or poor understanding of personal risk of disease. We developed an online CRC risk assessment (http://www.clevelandclinic.org/score) incorporating family and personal history of adenomas and CRC which generated a pedigree, risk category and screening recommendations based on ACG guidelines. Modifiable lifestyle factors were also assessed and personalized recommendations were provided to minimize neoplasia due to those factors. We assessed the feasibility and online uptake of this tool and determined the proportion of high risk individuals who meet criteria suspicious for HNPCC.

## Methods

The assessment included questions on demographics, use of previous CRC screening, and family and personal history of adenomas and CRC in 3 generations. Height, weight, age > or < 50, race, smoking exposure, physical activity, and dietary habits assessed. Risk categories included average, low, medium, and high.

## Results

3515 participants completed the assessment. 67% male, 81% white and 46% were < age 50 with mean BMI of 28.4. 53% reported eating < 3 servings of fruits/vegetables daily and 45% didn’t exercise > 30 minutes > 3 days/week. 61% never smoked and 28% were former smokers. 39% reported previous screening; 89% utilizing colonoscopy. 11% reported a history of adenomas and 1.3% CRC. The 405 individuals who reported a personal history of adenomas/CRC had a higher BMI 29.6 vs 28.1 (p= 0.013); age > 50 19% vs 6% (p < 0.001); greater current packs of cigarettes (p = 0.024) and years of current smoking (p = 0.032) than those without adenomas/CRC. Self reported screening use was associated with increased intake of fruits/vegetables (3-5 vs < 3 servings daily 1.8 (1.4-2.3, p < 0.0001), FDR with CRC 2.2 (1.6-3.2, p < 0.0001) and sibling with CRC 4.2 (2.3-7.7, p = 0.0001). The risk category (Table 1) revealed that 65% were average, 11% low, 22% medium, and 2% high risk. This prevalence of high risk individuals is similar to the frequency of Lynch syndrome reported in unselected CRC populations.

**Table 1:** Risk Factors, Risk Category and Screening Recommendation

No personal hx of CRC or polyps & no FDR with CRC or polyps	Average Risk	Colonoscopy every 10 yrs beginning age 50 (or 45 if black)
1 FDR with CRC or polyps age ≥ 60	Low Risk	Colonoscopy every 10 yrs beginning age 50 (or 45 if black)

Personal history of CRC or adenomatous polyps FDR with CRC or adenomatous polyps < 60 yrs or in ≥ 2 FDR at any age	Medium Risk	Colonoscopy at an interval dependant on size, number and pathology of previous polyps Colonoscopy every 5 years beginning age 40 or 10 years before youngest age of relative affected, whichever is earlier

Combinations of 3 individuals with CRC which may include self, and ≥ 1 FDR and/or SDR (same side of family)	High Risk	Suspicious for hereditary CRC syndrome. Risk assessment by specialists skilled in genetic assessments. Early onset colonoscopy ± genetic testing

## Conclusion

An online risk assessment is feasible to provide education regarding CRC risks and screening recommendations. It appears useful to identify individuals at risk of HNPCC although validation of the reported history is required.

